# Further characterization of autoantibodies to GABAergic neurons in the central nervous system produced by a subset of children with autism

**DOI:** 10.1186/2040-2392-2-5

**Published:** 2011-04-26

**Authors:** Sharifia Wills, Christy C Rossi, Jeffrey Bennett, Veronica Martinez-Cerdeño, Paul Ashwood, David G Amaral, Judy Van de Water

**Affiliations:** 1 Division of Rheumatology, Allergy and Clinical Immunology, University of California at Davis, 451 Health Sciences Drive, Suite 6510 GBSF, Davis, CA 95616, USA; 2 Department of Psychiatry and Behavioral Sciences, Center for Neuroscience, California National Primate Research Center, University of California, Davis, Davis, CA 95616, USA; 3 Department of Medical Microbiology and Immunology, University of California at Davis, Davis, CA 95616, USA; 4 The M.I.N.D. Institute, University of California Davis Medical Center, Sacramento, CA 95817 USA; 5 NIEHS Center for Children's Environmental Health, University of California, Davis, Davis, CA 95616, USA

## Abstract

**Background:**

Autism is a neurodevelopmental disorder characterized by impairments in social interaction and deficits in verbal and nonverbal communication, together with the presence of repetitive behaviors or a limited repertoire of activities and interests. The causes of autism are currently unclear. In a previous study, we determined that 21% of children with autism have plasma autoantibodies that are immunoreactive with a population of neurons in the cerebellum that appear to be Golgi cells, which are GABAergic interneurons.

**Methods:**

We have extended this analysis by examining plasma immunoreactivity in the remainder of the brain. To determine cell specificity, double-labeling studies that included one of the calcium-binding proteins that are commonly colocalized in GABAergic neurons (calbindin, parvalbumin or calretinin) were also carried out to determine which GABAergic neurons are immunoreactive. Coronal sections through the rostrocaudal extent of the macaque monkey brain were reacted with plasma from each of seven individuals with autism who had previously demonstrated positive Golgi cell staining, as well as six negative controls. In addition, brain sections from adult male mice were similarly examined.

**Results:**

In each case, specific staining was observed for neurons that had the morphological appearance of interneurons. By double-labeling sections with plasma and with antibodies directed against γ-aminobutyric acid (GABA), we determined that all autoantibody-positive neurons were GABAergic. However, not all GABAergic neurons were autoantibody-positive. Calbindin was colabeled in several of the autoantibody-labeled cells, while parvalbumin colabeling was less frequently observed. Autoantibody-positive cells rarely expressed calretinin. Sections from the mouse brain processed similarly to the primate sections also demonstrated immunoreactivity to interneurons distributed throughout the neocortex and many subcortical regions. Some cell populations stained in the primate (such as the Golgi neurons in the cerebellum) were not as robustly immunoreactive in the mouse brain.

**Conclusions:**

These results suggest that the earlier report of autoantibody immunoreactivity to specific cells in the cerebellum extend to other regions of the brain. Further, these findings confirm the autoantibody-targeted cells to be a subpopulation of GABAergic interneurons. The potential impact of these autoantibodies on GABAergic disruption with respect to the etiology of autism is discussed herein.

## Background

Autism is a lifelong neurodevelopmental disorder that is diagnosed in early childhood and is characterized by a core deficit in social interaction with impairments in communication, stereotypical movements and restricted behaviors [1]. Converging evidence over the past 40 years indicates that immune dysfunction may be an important factor contributing to the development of a subset of cases of autism [2-4]. Several studies have shown peripheral immune abnormalities in patients with autism [5-7]. There is provocative evidence for an ongoing inflammatory response in some individuals with autism [8,9].

The possibility has been raised that some forms of autism may be due to an autoimmune process [10]. There are a number of precedents for autoimmune diseases of the central nervous system. The best known among these are multiple sclerosis [11,12] and Sydenham's chorea [13,14]. The potential for an autoimmune etiology with respect to psychiatric disorders, including the pediatric autoimmune neuropsychiatric disorders associated with streptococcal infections, remains intriguing but controversial [14].

A number of reports have identified antibodies in individuals with autism that are directed against several central nervous system proteins. These include glial and neuron-axon filament proteins [15] (but see [16]), myelin basic protein [17] (but see [18]), serotonin receptor [19], nerve growth factor [20], cerebellar peptides [21], brain-derived neurotrophic factor [22], brain endothelial cells [23] and the caudate nucleus [24]. We have described an increased incidence of autoantibodies to brain proteins in autism compared with controls [25].

In a previous study [26], we used Western blot analysis and tissue immunohistochemistry to investigate the presence of autoantibodies to cerebellar tissue. Using Western blot analysis, we found that the plasma of 21% of individuals with autism demonstrated immunoreactivity to a protein of approximately 52 kDa from the human cerebellum that was present in only 2% of typically developing controls. When these plasma samples were then used as primary antibody sources for tissue immunohistochemistry using sections from the macaque monkey brain, 21% of the samples from children with autism spectrum disorder (ASD) compared to 0% of typically developing controls demonstrated intense immunoreactivity to what were morphologically determined to be Golgi cells of the cerebellum. This study utilized sections from the cerebellum based on early data that the cerebellum was preferentially involved in autism. However, it is now clear that autism affects many brain regions [27], and the question arises whether the autoantibodies present in autistic individuals identify a broader class of neurons that are distributed throughout the brain. Thus, to build upon our previous findings, in the current study we used plasma from the same individuals who demonstrated positive cerebellar Golgi cell immunoreactivity to stain tissue sections from the full rostrocaudal extent of the macaque monkey brain. To evaluate conservation of the target antigens, we also examined brain sections from adult male mice. We were particularly interested in determining whether the autoantibody immunoreactivity would identify neurons from disparate brain regions and whether the identified neurons would have any unifying morphological features.

## Methods

### Participants and sample collection

The study protocol followed the ethical guidelines of the most recent Declaration of Helsinki [28] and was approved by the Institutional Review Board of the University of California-Davis School of Medicine. All participants enrolled in the study had written informed consent provided by their parents and provided consent to participate if developmentally able. Subjects for this study were enrolled through the Medical Investigations of Neurodevelopmental Disorders (M.I.N.D.) Institute clinic. The current study was designed to be a detailed follow-up of our previous study that described the presence of Golgi cell immunoreactivity in the plasma of a subpopulation of children with autism [26]. Our current study population consisted of a subgroup of children previously diagnosed on the autism spectrum (*n *= 7) (age range, 2.5 to 7 years) who showed reactivity to an approximately 52-kDa protein in the cerebellum as determined by Western blot analysis as well as on the basis of intense immunoreactivity to Golgi cells of the cerebellum in the cynomolgus monkey (*Macaca fascicularis*). We also examined seven participants from our previous study who were diagnosed on the autism spectrum but did not show immunoreactivity to monkey brain sections. Our control population consisted of typically developing children with an absence of reactivity to both the 52-kDa protein and to neurons of the cynomolgus monkey brain (*n *= 6) (age range, 2.5 to 8 years). Any participants in the control group who were above criteria in either the Autism Diagnostic Interview-Revised (ADI-R) or the Autism Diagnostic Observation Schedule (ADOS) [1,29-31] were excluded. Samples from children with ASD with an absence of reactivity detected by both Western blot analysis and immunohistochemical staining were also examined. A diagnosis of ASD was confirmed in all participants on the basis of the ADI-R and ADOS score. The final ASD status is defined as meeting criteria on the communication, social interaction and repetitive behavior domains of the ADI-R with onset prior to 36 months of age and scoring at or above the social plus communication cutoff for autism on the ADOS module 1 or 2. The Social Communication Questionnaire [32] was used to screen for characteristics of ASD among the typically developing controls. Children who scored above the screening cutoff were fully assessed using the ADI-R and ADOS.

### Animals and fixation

#### Nonhuman primate tissue

We choose nonhuman primate tissue as our antigen source because of the highly conserved nature of autoantigens as well as the similar but compressed neurodevelopment of rhesus monkeys compared with humans [33]. All procedures were carried out under an approved University of California-Davis Institutional Animal Care and Use Protocol and strictly adhered to National Institutes of Health policies on primate animal subjects. The brain sections were obtained from two different male adult cynomolgus macaques (*Macaca fascicularis*): one was 9 years old and the other was 15 years, 4 months old at the time they were killed. The brains from these animals had been used for neural tract tracing studies, and library sections were used for the present study. No animals were killed expressly for these studies. The sections were obtained from animals for which no health problems were reported.

Tissue fixation and histological processing were performed according to procedures described previously [34]. Briefly, the animals were deeply anesthetized and perfused intracardially with 1% paraformaldehyde in 0.1 M phosphate buffer (pH 7.4) at 4°C at a rate of 250 mL/minute for 2 minutes, followed by 4% paraformaldehyde in 0.1 M phosphate buffer (pH 7.4) at 4°C at a rate of 250 mL/minute for 10 minutes. The flow rate was then reduced to 100 mL/minute for 50 minutes. The animal was then perfused with 5% sucrose in 0.1 M phosphate buffer (pH 7.4) to remove as much fixative as possible.

The brain was blocked stereotaxically, extracted from the skull and cryoprotected in a solution containing 10% glycerol and 2% dimethyl sulfoxide (DMSO) for 1 day, followed by 20% glycerol and 2% DMSO for 3 days. The fixed brain was then frozen using the isopentane method [35] and stored at -70°C until sectioning. Frozen sections were cut in the coronal plane at a thickness of 30 μm with a sliding microtome and placed into a cryoprotectant tissue-collecting solution (30% ethylene glycol, 25% glycerin in 0.05 M sodium phosphate buffer). The sections were stored at -80°C until they were used for immunohistochemistry.

Additionally, tissue from one adult rhesus macaque (*Macaca mulatta*) whose perfusion included glutaraldehyde was used for anti-γ-aminobutyric acid (anti-GABA) immunostaining. Briefly, the perfusion consisted of 0.9% sodium chloride at a rate of 250 mL/minute for 2 minutes, followed by 4% paraformaldehyde/0.1% glutaraldehyde at a rate of 250 mL/minute and finally 4% paraformaldehyde/0.1% glutaraldehyde at a rate of 100 mL/minute for 50 minutes. Tissue was blocked and sectioned in a manner similar to that described for the cynomolgus macaques.

### Mouse tissue

Two adult male C57BL/6 mice were perfused according to the methods described above for the cynomolgus macaque tissue, with an initial flow rate of 6 mL/minute followed by a flow rate of 4.75 mL/minute to account for the animal's smaller size. Tissue was cryoprotected in 10% glycerol and 2% DMSO for 1 day, followed by 20% glycerol and 2% DMSO for 2 days, and was sectioned on a sliding microtome at 30 μm as previously described for the monkey tissue.

### Immunohistochemistry

Free-floating macaque brain sections were processed using methods employed and described previously [34,36,37]. Serial sections were used for immunohistochemistry using samples from each subject individually. Briefly, 30-μm-thick coronal sections from the brain were taken from storage, placed into nets and then washed (3 × 10 minutes with 0.1 M phosphate-buffered saline (PBS)). The following steps were followed with agitation at room temperature, except for the primary incubation, which was performed at 4°C with agitation. Prior to primary incubation, the sections were pretreated with hydrogen peroxide (1.6% hydrogen peroxide in 0.1 M PBS (Fisher Scientific; Pittsburg, PA, USA) for 15 minutes to remove endogenous peroxidases and reduce background staining. During incubations, the tissue was maintained in solution at all times. The sections were blocked for 4 hours using 5% normal mouse serum (Pierce Biotechnology; Rockford, IL, USA) in 0.1 M PBS and 0.5% Triton X-100 (Fisher Scientific; Pittsburg, PA, USA). The nets were incubated with the primary antibody (subject plasma) at a dilution of 1:100 in 0.1 M PBS, 2% normal mouse serum and 0.3% Triton X-100 for 40 to 48 hours. The sections were then washed with 2% normal goat serum (Invitrogen; Carlsbad, CA, USA) in 0.1 M PBS (3 × 10 minutes) and incubated with biotinylated mouse anti-human monoclonal immunoglobulin G (IgG) (Zymed: Carlsbad, CA, USA) in 0.1 M PBS, 2% normal mouse serum and 0.3% Triton X-100 for 1 hour. Sections were then washed with 2% normal goat serum in 0.1 M PBS (3 × 10 minutes) followed by incubation for 45 minutes with avidin-biotin-complex (ABC) peroxidase (Biostain Super ABC/Peroxidase Basic Kit; Biomeda: Foster City, CA, USA) incubation in 0.1 M PBS. Following the first ABC peroxidase incubation, the sections were washed in 2% normal goat serum in 0.1 M PBS. The incubation with the secondary antibody (biotinylated mouse anti-human monoclonal antibody) was repeated for 45 minutes, followed by washes with 0.1 M PBS only. The second ABC peroxidase incubation was for 30 minutes, followed by washes with 0.05 M Tris buffer. Next, the sections were incubated with 3,3-diaminobenzidine (DAB) (Fisher Scientific) in 0.05 M Tris with 0.04% hydrogen peroxide for 30 minutes. The sections were then washed twice with 0.05 M Tris, once with 0.02 M potassium PBS (KPBS) and stored covered at 4°C in 0.02 M KPBS. Sections were then mounted from phosphate buffer onto gelatin-coated slides and dried with a fan, then placed in a 37°C incubator for 24 hours to dry. Mouse sections were processed in an identical manner, except for the substitution of 0.5% for 1.6% hydrogen peroxide and the additional use of an avidin and biotin blocking kit (Vector Laboratories; Burlingame, CA, USA) prior to incubation in the primary antibody solution.

### Silver nitrate/gold chloride intensification

To increase the signal-to-noise ratio of the immunohistochemical reaction product, a silver nitrate/gold chloride intensification procedure was followed for immunohistochemical analysis for those sections stained with DAB. Intensification was not used for sections visualized by using fluorescence immunohistochemistry. Prior to intensification, the slides were defatted in a mixture of equal parts chloroform and 100% ethanol for a total of 4 hours. The sections were then hydrated through graded ethanols and placed in a 37°C incubator overnight. Following the overnight incubation, the slides were rinsed with running, deionized water for 10 minutes, followed by incubation in a 1% silver nitrate solution maintained at 56°C with the use of a water bath for 40 minutes. The slides were then rinsed with running, deionized water for 10 minutes and incubated for 10 minutes in a 0.2% gold chloride solution with agitation at room temperature. Following a second rinse, the slides were stabilized in a 5% sodium thiosulfate solution at room temperature for 15 minutes with agitation, followed by an additional rinse in running, deionized water for 10 minutes. Sections were then dehydrated through graded ethanols, followed by xylenes, and coverslipped using DPX mounting medium (Sigma Aldrich; St. Louis, MO, USA).

Slides were observed by bright-field microscopy on a Nikon Eclipse E600 (Nikon Americas; Melville, NY, USA) or a Leica Leitz DMRB microscope (Leica Microsystems; Buffalo Grove, IL, USA) and photographed using the Spot Diagnostic Instruments Digital Camera System and software (Spot Imaging Solutions; Sterling Heights, MO, USA). Preliminary identification of the various cell types was accomplished using morphological features such as their location, cell body size and dendritic configuration.

### Fluorescence immunohistochemistry

Since it appeared that the majority of neurons that demonstrated specific immunoreactivity when incubated with plasma from individuals with autism had the morphological characteristics of GABAergic neurons, we carried out analyses designed to confirm this finding. First, we compared the distribution of the plasma-stained neurons with libraries of sections stained immunohistochemically for the distribution of glutamic acid decarboxylase 65 (GAD65) and GAD67, GABA or *in situ *hybridization with probes to GABAergic neurons [34,37]. Second, we carried out studies in which sections were double-labeled for the presence of plasma-identified interneurons and GABA immunoreactivity. Finally, we double-labeled additional sections for plasma-labeled interneurons and the calcium-binding proteins calbindin, parvalbumin and calretinin.

For double-labeling with plasma and anti-GABA, sections from the glutaraldehyde-fixed macaque brain were rinsed in PBS, then blocked in 0.2% gelatin, 1% Triton X-100 and 5% normal donkey serum in 0.1 M PBS for 2 hours, and incubated with plasma from an individual with autism as well as monoclonal mouse anti-GABA diluted 1:400 (a gift from Ismo Virtanen) at 4°C for 72 hours. Tissue was then rinsed and incubated in Texas Red-AffiniPure donkey anti-human IgG (1:100; Jackson ImmunoResearch; West Grove, PA, USA) and fluorescein isothiocyanate-conjugated horse anti-mouse IgG (1:500; Vector Laboratories) for 2 hours at 4°C. For double-labeling with plasma and antibodies directed to calcium-binding proteins (calbindin, parvalbumin or calretinin), a Tyramide Signal Amplification (TSA) kit (PerkinElmer; Waltham, MA, USA) was used to enhance the fluorescent signal obtained from labeling with plasma. Sections were first rinsed in 0.1 M PBS, pretreated with 1% hydrogen peroxide in PBS for 30 minutes, rinsed in 0.1 M PBS and then blocked with 0.5% Triton X-100 and 5% normal mouse serum in 0.1 M PBS for 4 hours. They were then incubated overnight with a 1:100 dilution of human plasma in 0.1% Triton X-100 and 1% normal mouse serum in PBS. Sections were subsequently rinsed in PBS and incubated in Tris-NaCl-Tween buffer (TNT) buffer for 15 minutes, followed by a 30-minute incubation in TNB buffer (Blocking buffer supplied with TSA kit; 0.1 M TRIS-HCl, pH 7.5. 0.15 M NaCl. 0.5% Blocking Reagent), and incubated in a 1:1,000 dilution of biotinylated anti-human IgG secondary (Zymed; Carlsbad, CA, USA) for 1 hour. The secondary antibody was washed away with TNT buffer, and sections were then incubated in streptavidin-horseradish peroxidase (included in the TSA kit) diluted 1:100 in TNB buffer for 30 minutes. After being washed with TNT buffer, sections were incubated in tetramethylrhodamine tyramide (PerkinElmer) diluted 1:50 in amplification diluent for 8 minutes at room temperature, washed in 0.1 M PBS and incubated overnight in a solution containing an antibody against calbindin (Swant; CH-1723 Marly 1, Switzerland), parvalbumin (Swant) or calretinin (Swant) diluted 1:1,000 in 0.02% gelatin, 0.1% Triton X-100 and 1% donkey serum in 0.1 M PBS. The next day sections were rinsed in 0.1 M PBS, incubated in secondary antibody (donkey anti-rabbit) diluted 1:200 in 0.1% Triton X-100 and 1% donkey serum in PBS for 2 hours, rinsed in PBS again and mounted onto slides and coverslipped with VECTASHIELD HardSet Mounting Medium (Vector Laboratories). Fluorescently labeled sections were examined and photographed using an Olympus FluoView confocal laser-scanning microscope, and analysis was performed using FluoView version 3.3 software (Olympus: Hamburg, Germany). For identification of labeled cells, we used the same criteria described for sections processed with diaminobenzidine.

## Results

### General staining pattern

A series of approximately 10 coronal sections from the *Macaca fascicularis *brain were processed for each of the plasma samples evaluated in this study. Sections were selected to obtain a sample from each of the following regions: frontal lobe, striatum, amygdaloid complex, hippocampal formation, hypothalamus, thalamus, cerebellum and primary visual cortex (V1). Because the complete coronal sections were processed, many additional cortical and subcortical regions, such as the thalamus, were also available for analysis. Neurons immunoreactive for IgG autoantibodies from individuals with autism were observed in all brain regions. Regardless of the region studied, the neurons tended to be small and to have the appearance of GABAergic interneurons. To provide a description of the types of staining patterns we observed, we have selected a few regions to describe in some detail. In all cases, the staining that we noted was consistently observed for all of the plasma samples from children with autism that demonstrated staining of Golgi cells in the cerebellum. We did not see this pattern of staining in any of the plasma samples from children with autism who did not demonstrate Golgi cell staining or in plasma samples from age-matched typically developing children from this study population.

### Cerebellum

**Figure 1:**
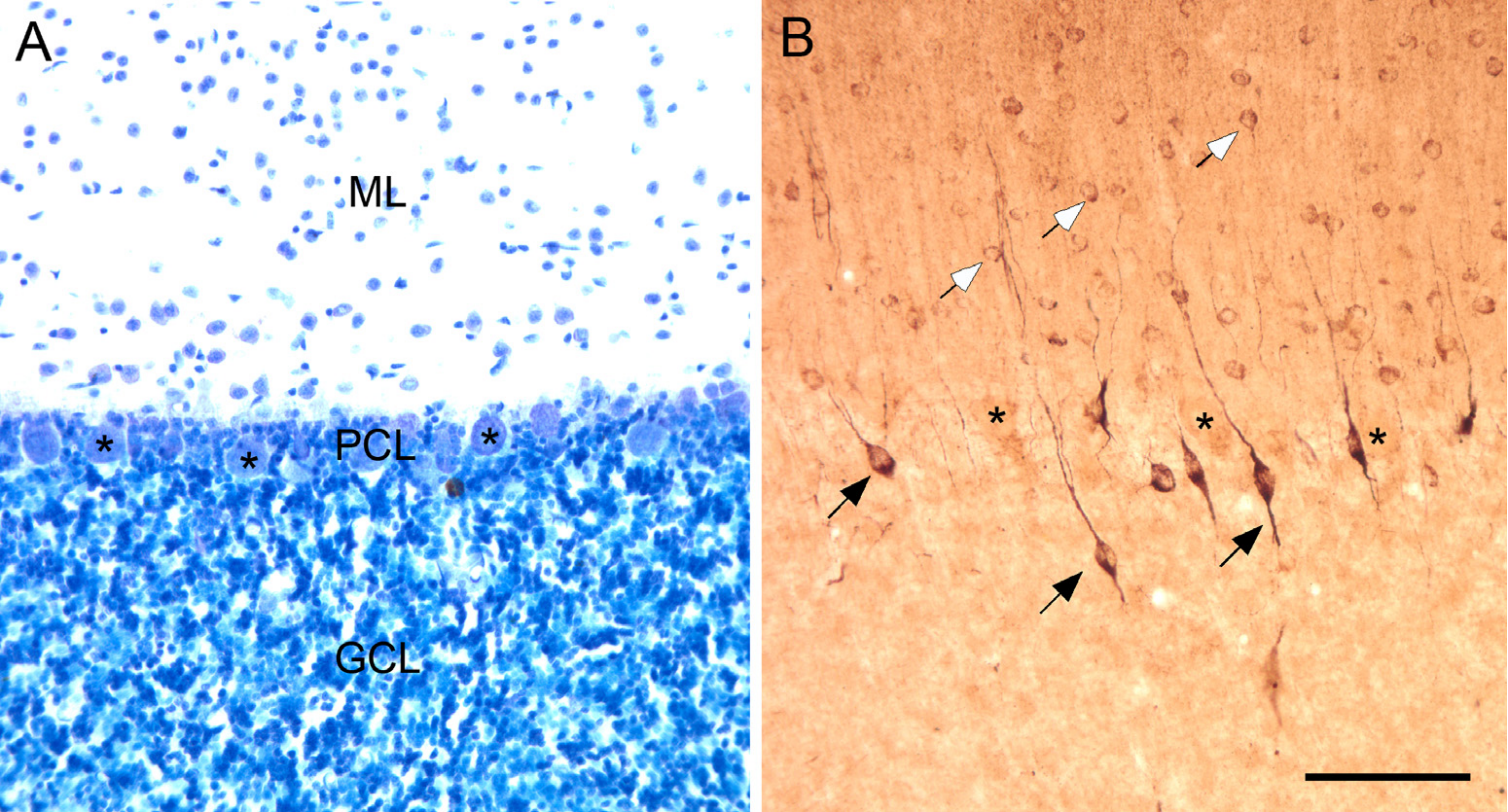
**Photomicrographs illustrating coronal sections through the macaque monkey cerebellum**. **(A) **Nissl-stained section illustrating the three major layers of the cerebellum: the granule cell layer (GCL), the Purkinje cell layer (PCL) and the molecular layer (ML). The locations of several large Purkinje cells (asterisks) are shown. **(B) **Immunoreactivity resulting from exposure of adjacent section to plasma from a representative child with autism (age 4 years). Golgi neurons (black arrows) are clearly labeled and lie adjacent to or below the Purkinje cell layer. Ghost images of Purkinje cells are marked with asterisks. Lighter, though consistent, labeling of basket cells (white arrows) was also observed with this plasma. Only plasma from subjects that provided this level of staining of the Golgi cells in the cerebellum was used for analysis of other brain regions. Calibration bar, 100 μm.

We previously showed that 21% of plasma samples taken from children with autism demonstrated selective, intense immunoreactivity against the Golgi cells of the cerebellum (Figure 1). In many cases, populations of basket cells in the molecular layer demonstrated specific, albeit less intense, immunoreactive staining. There were no other cell types in the cerebellar cortex that were recognized by these IgG autoantibodies.

### Cerebral cortex (area V1)

**Figure 2:**
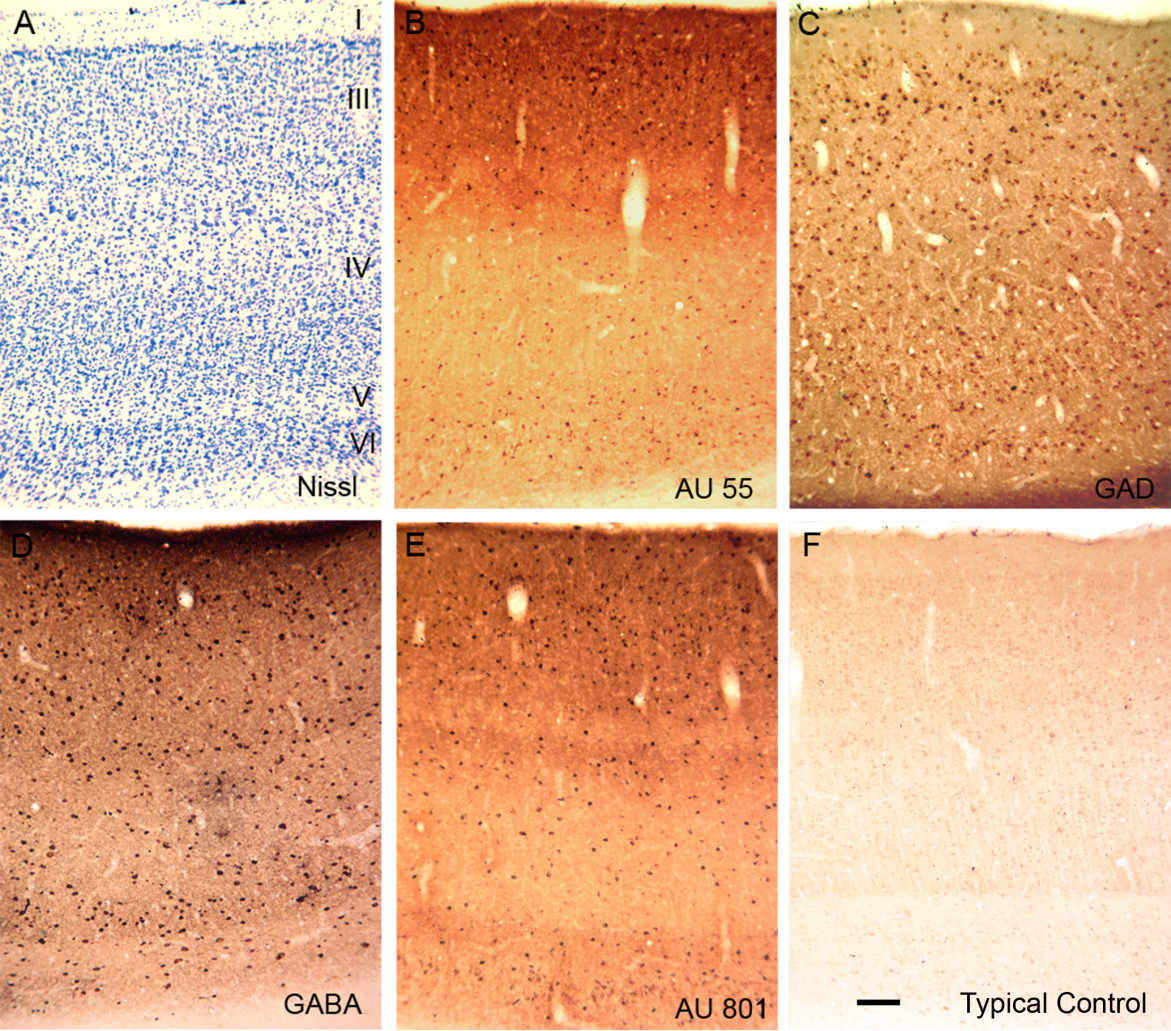
**Photomicrographs illustrating coronal sections through primary visual cortex (V1) of the macaque monkey brain**. **(A) **Nissl-stained section showing the lamination pattern (cortical layers I to VI) of neurons in this region. **(B) **Staining of V1 with plasma from one child with autism (age 6 years). Note that in this panel and Figure 2E, the highest numbers of labeled neurons are located in the superficial layers (I to III). **(C) **Section through area V1 demonstrating *in situ *hybridization with a probe to glutamic acid decarboxylase 67 (GAD67). The brown reaction product illustrates neurons that are GABAergic. **(D) **Very similar representation of GABAergic neurons identified immunohistochemically with a monoclonal antibody to γ-aminobutyric acid (GABA). **(E) **Staining very similar to that shown in Figure 2B of plasma from another child with autism (age 5 years). **(F) **Section through area V1 that was reacted with plasma from a typically developing child (age 5 years). While there is light, nonspecific background staining that resembles the distribution of Nissl-stained cell bodies, there is no specific labeling of GABAergic neurons. Calibration bar, 100 μm.

We next focused our analysis of the cerebral cortex on area V1 of the occipital lobe (Figure 2). This cortical region was selected because of the nature of its well-characterized cellular neuroanatomy. The pattern of immunoreactive staining was consistent across all plasma samples (Figures 2B and 2E). While there was some heterogeneity in the morphology of labeled neurons, the vast majority of cells appeared to be small, circular cells with several radiating dendrites. Similar patterns were detected between the autoantibody-labeled cells and cells labeled by *in situ *hybridization with a probe to GAD67 (Figure 2C) or cells labeled immunohistochemically with an antibody to GABA (Figure 2D). The labeled cells had very similar morphology in all three preparations. The distribution of labeled cells was somewhat different, however. With the plasma autoantibodies, there was a clear preponderance of labeled neurons in the superficial layers (I to III), whereas fewer labeled cells were observed in the deep layers (IV to VI). For the GAD67 and GABA preparations, however, the distribution of labeled cells was more homogeneous across all of the cortical layers. In particular, it appeared that there were relatively few autoantibody-reactive cells in layer V in comparison to the GAD67- and GABA-labeled cells. This raised the prospect that the vast majority of neurons recognized by the autoantibodies from individuals with autism were GABAergic interneurons. However, it also appeared likely that these antibodies were not staining a population of GABAergic neurons located in the deep cortical layers.

**Figure 3:**
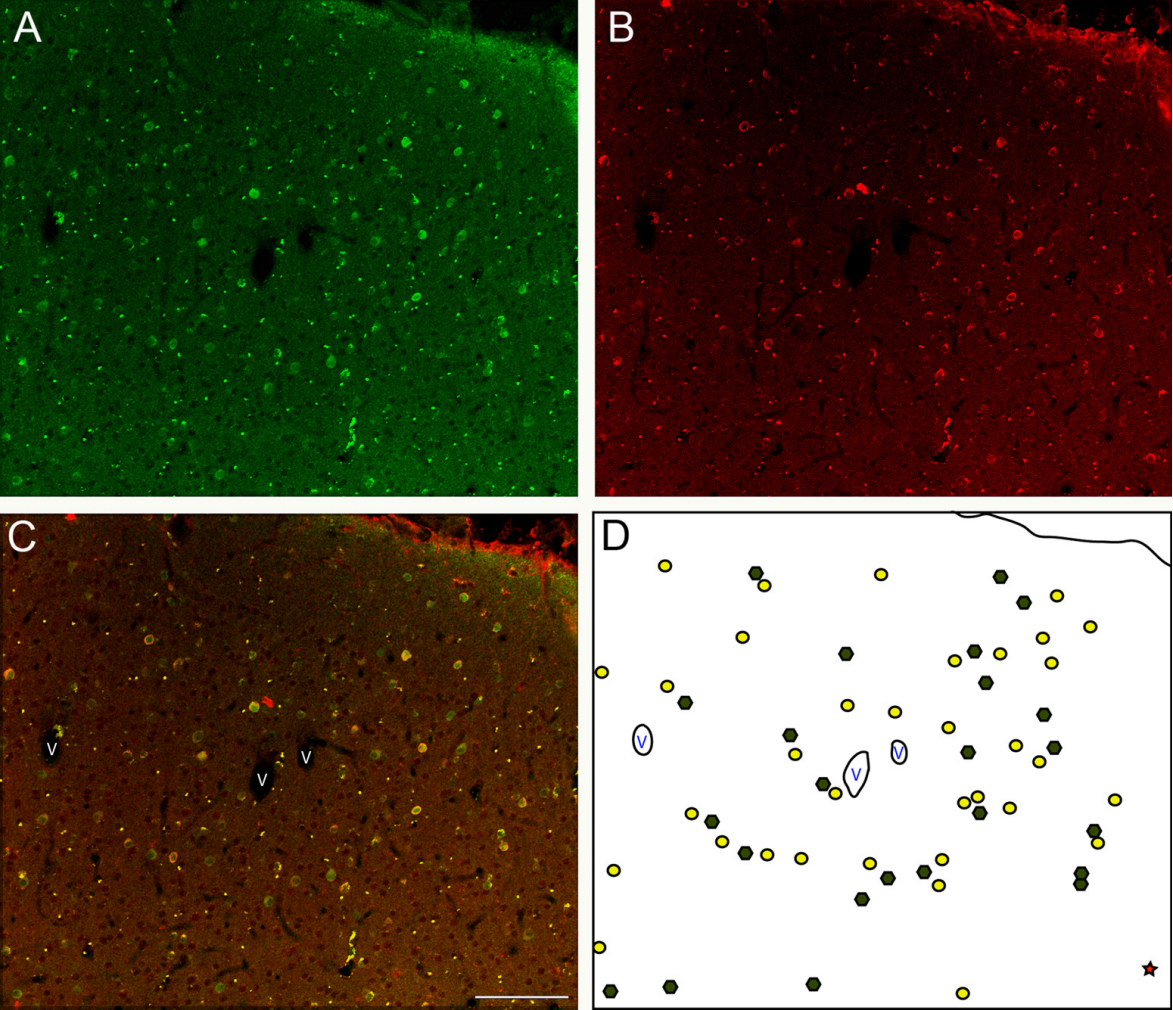
**Fluorescence confocal photomicrographs of double-labeling with plasma from a representative child with autism (age 5 years) and an antibody directed at GABA**. **(A) **GABA immunoreactivity in area V1 (green). **(B) **Immunoreactivity of V1 with plasma from a child with autism (red). **(C) **Merged images from Figures 3A and 3B showing several double-labeled cells (yellow). The vessels have been labeled (V) to aid in orientation with Figure 3D. **(D) **Schematic of the same region shown in Figures 3A to 3C, with cells coexpressing GABA and plasma immunoreactivity, depicted as yellow circles, GABAergic cells without plasma immunoreactivity, depicted as green hexagons, and a single GABA-negative, plasma-positive cell, depicted as a red star. The vessels have been labeled (V) to aid in orientation with Figure 3C. Calibration bar, 100 μm.

**Figure 4:**
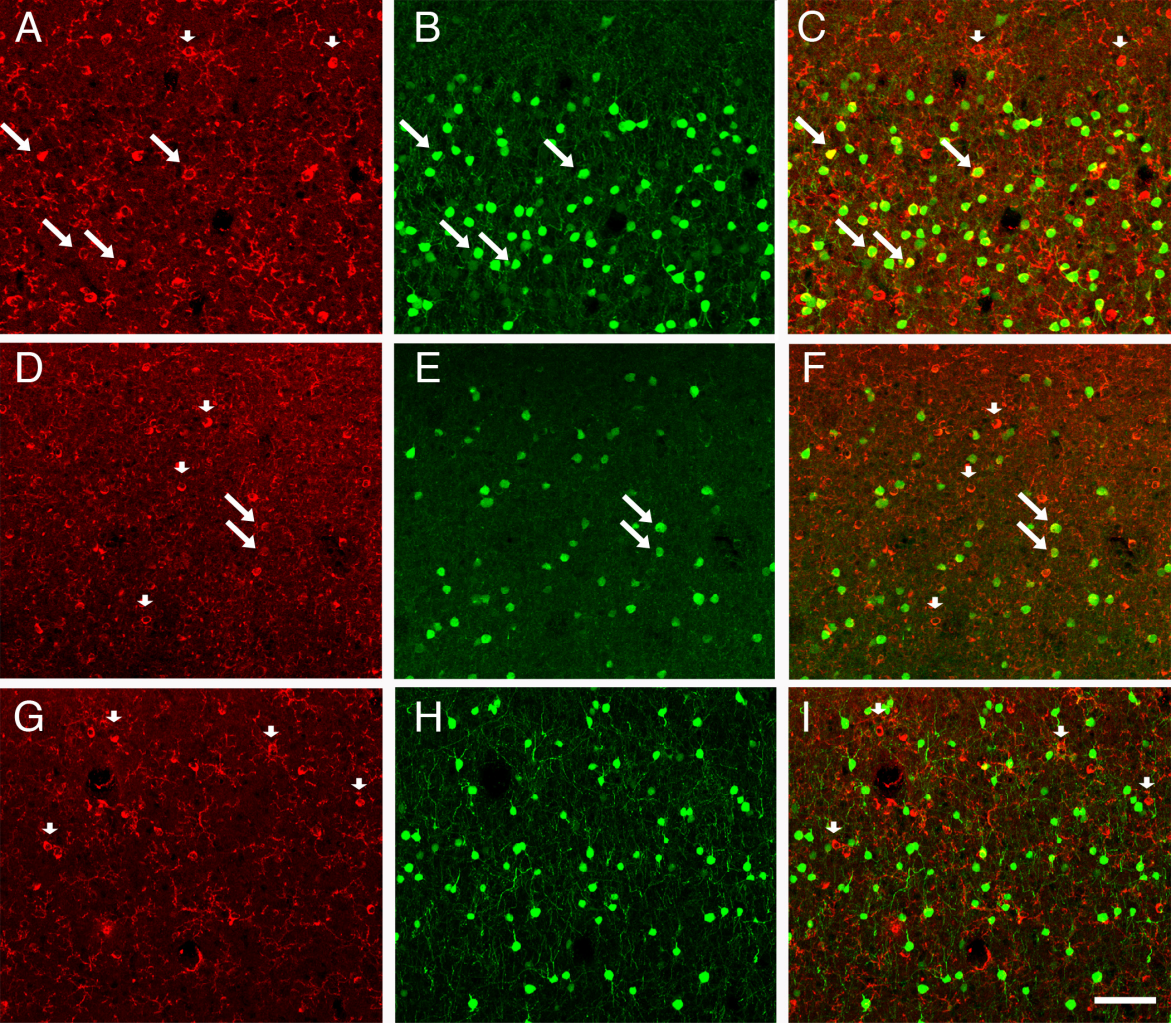
**Fluorescence confocal photomicrographs of double-labeling with plasma from a representative child with autism and antibodies directed toward calcium-binding proteins in area V1**. **(A to C) **Immunoreactivity of **(A) **the autism (AU)-specific autoantibodies, **(B) **calbindin and **(C) **the merged image showing that several cells are colabeled (arrows), but that not all AU immunoglobulin G (IgG)-labeled cells express calbindin (arrowheads). Double-labeling with plasma from **(D) **a autoantibody-positive child with autism and **(E) **anti-parvalbumin shows that some AU IgG-labeled cells express parvalbumin (arrows), while most do not (arrowheads). **(G to I) **AU immunoreactivity **(G) **rarely occurs in cells that express calretinin **(H) **with no co-localization evident **(I)**. Arrowheads point to cells that are labeled by the autoantibody, but not by anti-calretinin **(I)**. Calibration bar, 100 μm.

To provide evidence for the proposition that the plasma labeled cells were GABAergic, an additional series of sections through area V1 were double-labeled with plasma and with anti-sera to GABA (Figure 3). On the basis of evaluation using the confocal laser microscope, we confirmed that essentially all autoantibody-reactive cells were double-labeled for GABA. We also confirmed that a sizable number of GABA-positive neurons were not immunoreactive with the plasma. Many of these single-labeled GABAergic neurons were located in the deep layers of the cortex. Because only a subset of GABA-positive neurons was labeled by autoantibody-containing plasma, we wanted to get a sense of which populations of GABAergic neurons were being labeled. While it was beyond the scope of the current paper to do an exhaustive, quantitative analysis with double-labeling, we did double-label sections through area V1 with plasma and antibodies directed against the calcium-binding proteins calbindin, parvalbumin and calretinin. We determined that plasma-labeled cells were not exclusively labeled by any one of these proteins expressed in GABAergic neurons. Rather, it appears that the immunoreactive cells were most commonly labeled by calbindin, but not all calbindin-labeled cells were labeled by the autoantibodies (Figures 4A to 4C). Autoantibody-labeled cells less commonly expressed parvalbumin (Figures 4D to 4F), and labeling with calretinin in plasma-labeled cells was rare (Figures 4G to 4I).

### Cerebral cortex (posterior cingulate cortex)

**Figure 5:**
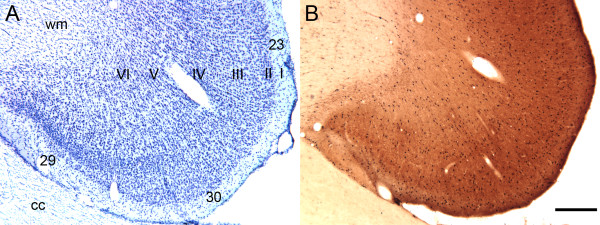
**Photomicrographs of coronal sections through the posterior cingulate cortex of the macaque monkey**. This level includes areas 29, 30 and 23. **(A) **Nissl-stained section showing the laminar pattern of posterior cingulate cortex located just dorsal to the corpus callosum (cc). The layers of cortical area 23 are marked I to VI. **(B) **Immunostaining with plasma from a representative child with autism. As in the primary visual cortex, the most numerous labeled neurons are located in the superficial layers (I to III), although immunopositive neurons are located throughout all layers. This pattern was recapitulated in all cortical areas. Labeled cells were not observed in the cc, although apparent positive cells were observed in the subcortical white matter. Calibration bar, 250 μm.

We have previously provided a detailed cytoarchitectonic analysis of the posterior and retrosplenial regions of the cingulate cortex [38]. Figure 5 shows a coronal section through this region that includes areas 29, 30 and 23. As in area V1, autoantibody-reactive cells are distributed throughout all layers of these cortical regions, with a slight predominance in superficial layers I to III. The white matter deep within these cortical areas (Figure 5B) has a mottled appearance. This is due to the labeling of a population of glial cells by the plasma. We describe these cells below.

### Hippocampal formation

**Figure 6:**
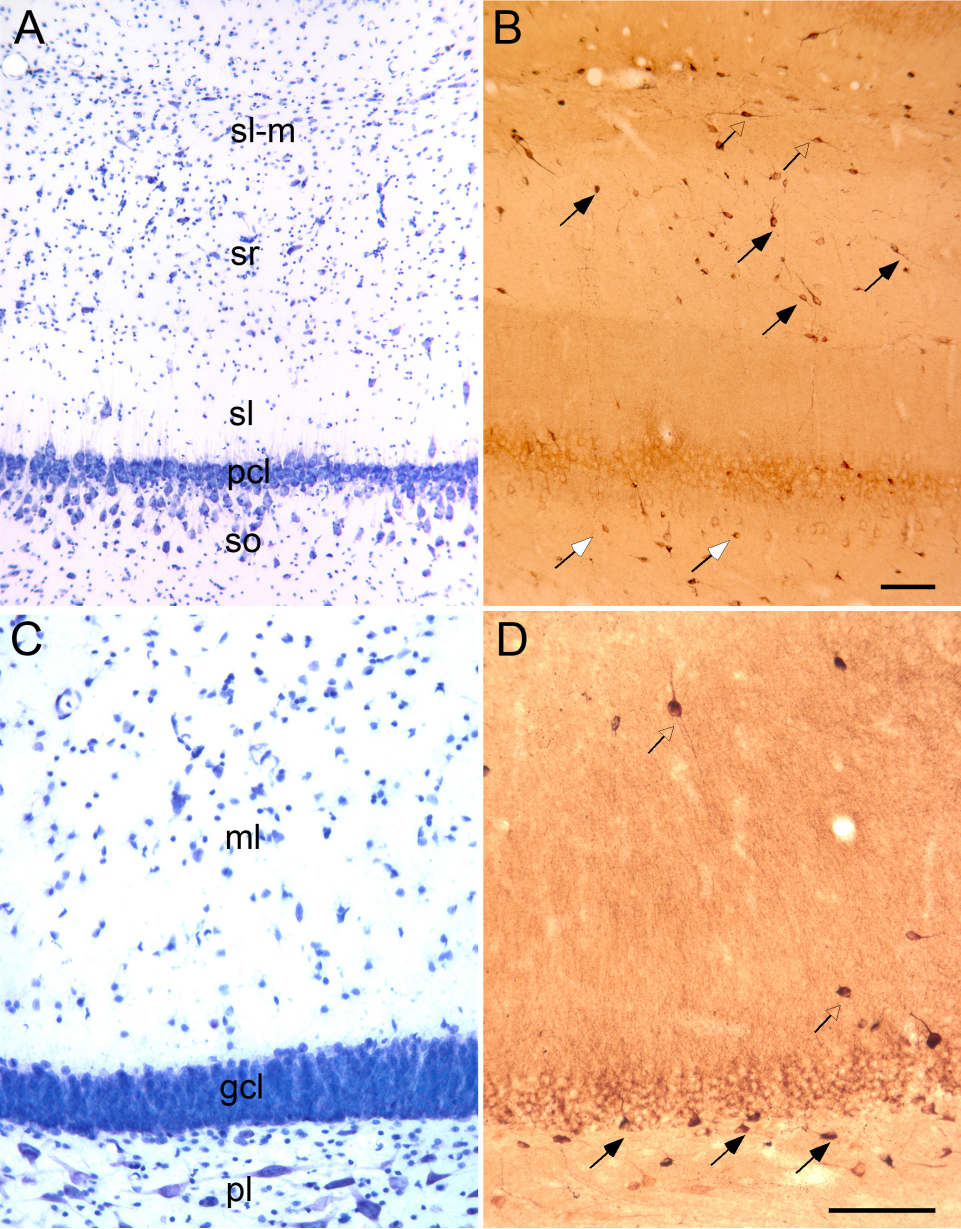
**Photomicrographs of the CA3 region of the hippocampus and the dentate gyrus of the macaque monkey**. **(A) **Nissl-stained section showing the major layers of the CA3 region of the hippocampus. Deep to superficial layers include so, stratum oriens; pcl, pyramidal cell layer; sl, stratum lucidum, sr, stratum radiatum, sl-m, stratum lacunosum-moleculare. **(B) **Section of the CA3 region of the hippocampus stained immunohistochemically with plasma from a representative AU subject. Numerous labeled neurons are observed in the stratum radiatum (black arrows) and the adjacent stratum lacunosum-moleculare (open arrows). Only background level staining is observed in the pyramidal cell layer, but there are a variety of labeled neurons in stratum oriens (white arrows). **(C) **Nissl-stained section showing the layers of the dentate gyrus. Layers include pl, polymorphic layer; gcl, granule cell layer; and ml, molecular layer. **(D) **Section of the dentate gyrus stained immunohistochemically with plasma from a child with autism. Numerous labeled neurons (black arrows) are shown in the polymorphic cell layer, whereas fewer positive cells (open arrows) are shown in the molecular layer. Calibration bars, 100 μm. The bar in B applies to A, and the bar in D applies to C.

As illustrated in Figure 6, there are populations of plasma-labeled cells in the hippocampus and dentate gyrus. We have previously evaluated the distribution of GABAergic neurons in the macaque monkey hippocampal formation [36,37]. The population of autoantibody-positive cells in the hippocampal formation was morphologically heterogeneous. In the CA3 region of the hippocampus (Figures 6A and 6B), labeled neurons were located mainly in the stratum oriens, stratum radiatum and stratum lacunosum-moleculare. Relatively few labeled neurons were observed in the pyramidal cell layer or the stratum lucidum. The dendrites of labeled neurons were well-stained and often had a horizontal orientation, particularly in stratum oriens and stratum lacunosum-moleculare. In the dentate gyrus (Figures 6C and 6D), the majority of labeled cells were located in the polymorphic layer. Again, they had variable morphologies. Some appeared to be typical dentate basket cells, but others had dendrites that were mainly oriented parallel to the granule cell layer. There were a few small, round, labeled cells in the molecular layer of the dentate gyrus.

### Amygdaloid complex

**Figure 7:**
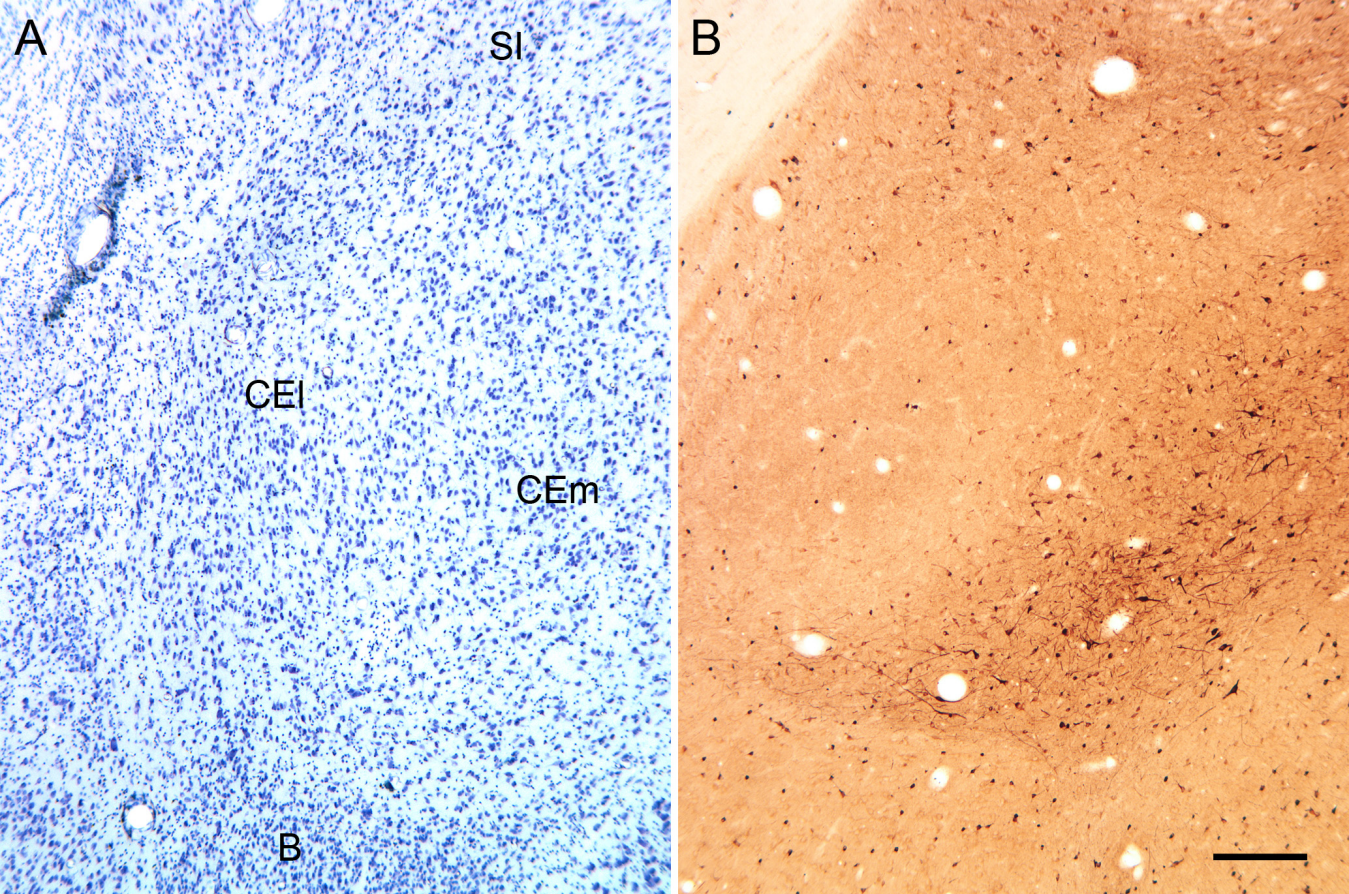
**Photomicrographs of the dorsal portion of the amygdala of the macaque monkey**. **(A) **Nissl-stained section of the dorsolateral portion of the amygdala. Much of the field is taken up by the central nucleus that can be divided into lateral (CEl) and medial (CEm)divisions. The basal nucleus **(B) **is located just ventral to the central nucleus. The substantia innominata (SI) is located just dorsal to the central nucleus. **(B) **Immunohistochemical staining of the amygdala using plasma from a representative child with autism. Labeled neurons are observed throughout all nuclei of the amygdala. In the central nucleus, the lateral nucleus has relatively few labeled neurons, whereas the density, size and dendritic staining of cells is much more prominent in the medial portion of the central nucleus. Calibration bar, 250 μm.

Autoantibody-labeled cells were distributed throughout all nuclei of the amygdala (Figure 7). Both the distribution and the appearance of the labeled neurons resembled GABAergic neurons based on our previous evaluation of GABA, GAD and parvalbumin labeling [34,37]. We have illustrated the distribution of autoantibody-labeled cells in and around the central nucleus of the amygdala (Figure 7). We had previously found that GABAergic neurons in the central nucleus do not stain well using antibodies directed against GABA [34]. However, *in situ *hybridization with probes to GAD65 or GAD67 demonstrated a very high density of GABAergic neurons spread more or less uniformly across the lateral and medial divisions of the nucleus. Interestingly, autoantibody labeling consistently demonstrated a high density of immunoreactive neurons with prominent dendritic staining located in the medial division of the nucleus. The lateral division, in contrast, demonstrated rather meager neuronal labeling reminiscent of the striatal labeling (see below).

### Striatum

**Figure 8:**
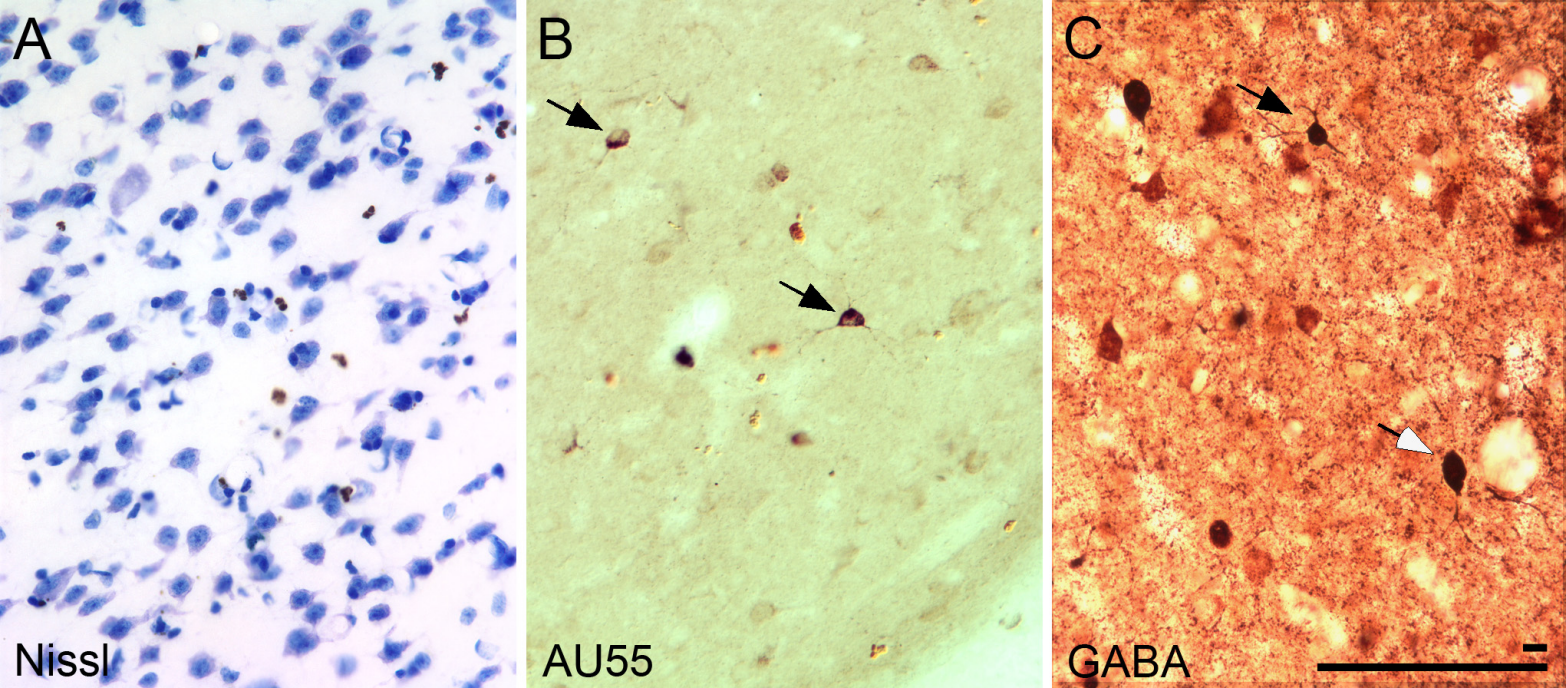
**Photomicrographs of the caudate nucleus of the macaque monkey**. **(A) **Nissl-stained section showing the density of striatal neurons in this region. **(B) **Phase-contrast photomicrograph illustrating small (approximately 10 μm in diameter), stellate neurons stained with plasma from a representative AU subject. **(C) **Section though the caudate nucleus reacted with a monoclonal antibody to GABA. Immunoreactivity is associated with numerous axonal varicosities as well as a variety of neuronal cell types. The small, stellate cell type observed by plasma immunohistochemical staining is also shown in the GABA preparations (black arrow). Other, larger GABAergic neurons (white arrow) can also be observed. Calibration bar, 10/100 μm.

We also observed autoantibody labeling within the caudate nucleus of the striatum (Figure 8), although there were very few labeled neurons in this region. All of the positively labeled cells were small (approximately 8 to 10 μm in diameter), with round cell bodies and short dendrites radiating in all directions (Figure 8B). When we examined sections stained immunohistochemically with an antibody to GABA (Figure 8C), it was clear that the autoantibody-labeled neurons resembled one of the classes of neurons demonstrating GABA immunoreactivity (Figure 8C). However, larger GABA-immunoreactive neurons that were not stained with the autoantibodies were also present.

### White matter

**Figure 9:**
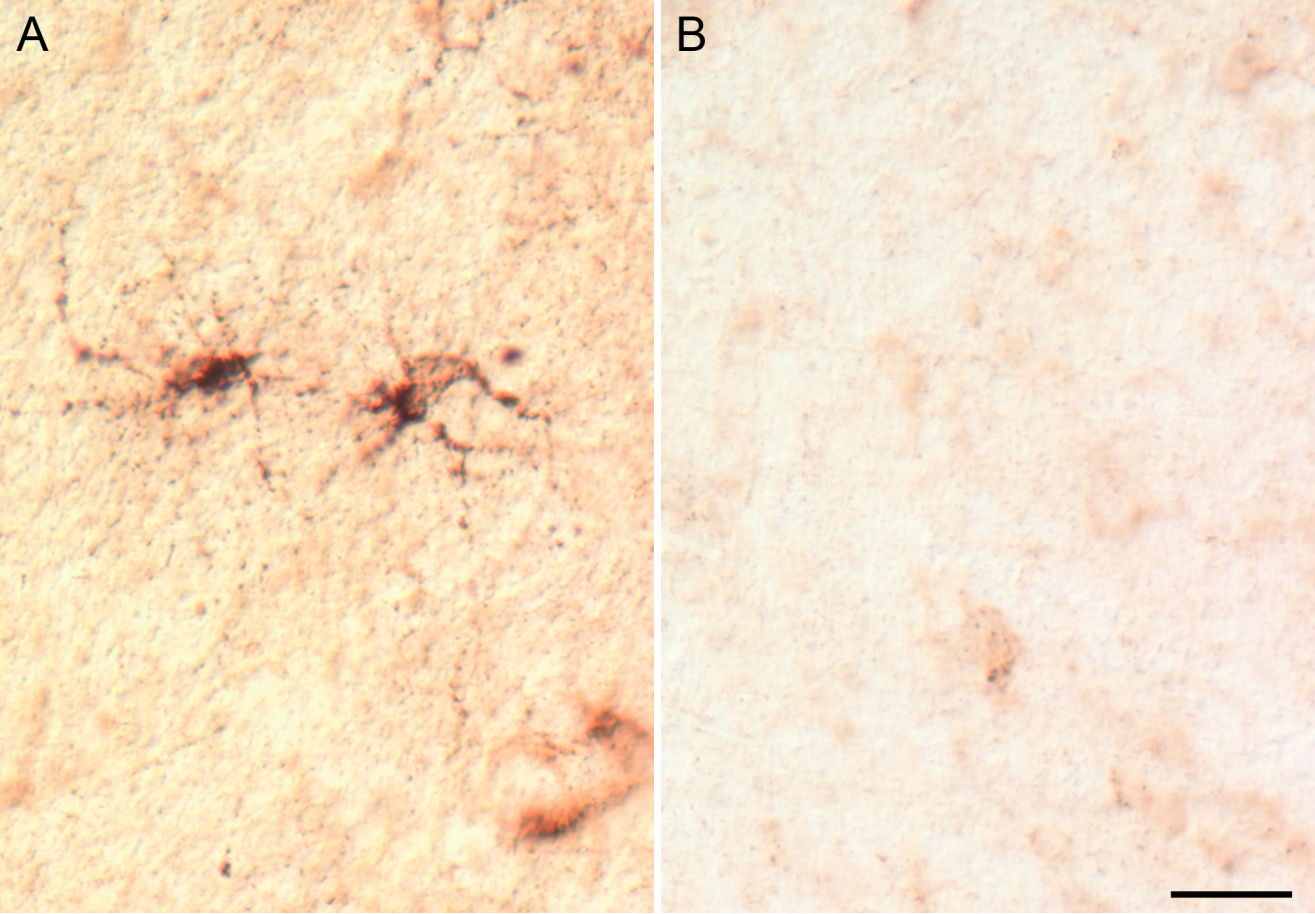
**Cellular profiles are also shown in the subcortical white matter in sections in which plasma stained GABAergic neurons**. **(A) **Phase-contrast photomicrograph of AU-immunoreactive, presumed microglial cells located in the white matter deep within the somatosensory cortex. **(B) **Similar region from a section reacted with plasma from a representative typically developing child in which no microglial labeling is observed. Calibration bar, 25 μm.

We did not observe any autoantibody-stained profiles within major fiber bundles such as the corpus callosum or the anterior commissure. However, in the white matter just subjacent to the neocortex, we did see reactive profiles that resembled microglial cells (Figure 9). Since we saw no evidence of astroglial staining in the cerebellum (Bergmann glial cells) or labeling of glial end-feet along the vasculature, these subcortical microglial cells appear to be the only non-neuronal cells immunoreactive with the autoantibodies. Interestingly, Butovsky *et al. *[39] recently demonstrated that exposure of microglia to the proinflammatory cytokine interferon-γ induced them to express both GABA and GAD67. Microglia also express GABA_B _receptors [40].

### Mouse tissue

**Figure 10:**
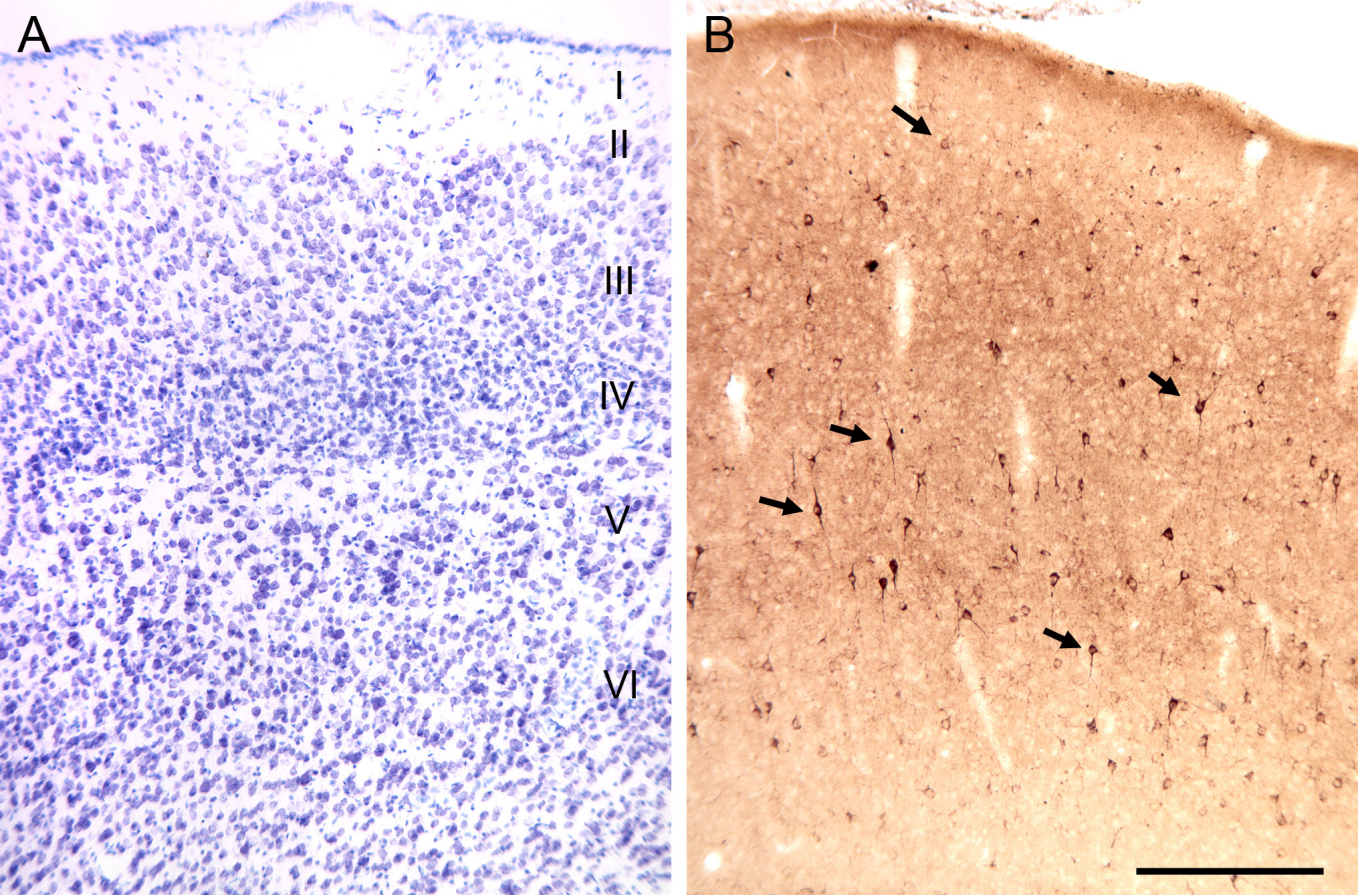
**Illustrations of plasma staining of the mouse somatosensory cortex**. **(A) **Nissl-stained section of the mouse somatosensory cortex. Layers I to VI are indicated on the right-hand side of the image. **(B) **Autoantibody staining of a section adjacent to the Nissl-stained section depicted in A with plasma from a representative AU subject. There are numerous immunoreactive neurons (arrows) scattered throughout all layers of the cortex. Calibration bar in B = 250 μm and applies to A as well.

Given the limited supply of plasma from the participants involved in this study, we were unable to carry out an exhaustive comparison of staining in the monkey and mouse brain. However, we were able to determine that the neurons that were morphologically characterized as interneurons were also labeled in various regions throughout the mouse brain. As illustrated in Figure 10, there were populations of immunoreactive neurons scattered throughout the neocortex. Many of these cells had substantial dendritic labeling (Figure 10D), and all resembled classes of interneurons. Immunoreactive neurons were observed in the hippocampus, amygdala and other brain regions that were also noted in the monkey brain (not shown). Interestingly, while the Golgi neurons of the mouse cerebellum were lightly immunoreactive, the intensity of staining was not nearly as striking as in the monkey brain or as intensely labeled as neurons observed in other brain regions. We intend to carry out a more exhaustive species comparison in a future study.

## Discussion

We previously reported that plasma from children with autism contains autoantibodies that are reactive to cerebellar Golgi cells [26]. The current study expands our earlier observations by exploring the pattern of immunohistochemistry in additional brain regions. We have demonstrated that the plasma from children with ASD contains antibodies that are reactive to neurons throughout the brain that are invariably GABAergic interneurons. We have also determined that not all GABAergic neurons are immunopositive, although we have not yet been able to determine in detail which categories of GABAergic neurons are positive for the plasma staining.

GABAergic cells form a heterogeneous population of neurons that are unevenly distributed in all layers of the monkey neocortex [41]. At least 20% of the neurons in the primary visual cortex are GABAergic interneurons [42], and the distribution in the monkey brain is quite similar to that in the human brain [43]. The plasma from children with autism allowed us to identify populations of cells in all layers of the cerebral cortex, with a slight preponderance in superficial layers. Colocalization studies using a monoclonal antibody to GABA revealed that the plasma from children with ASD appeared to recognize GABAergic interneurons in V1. There were fewer plasma-labeled cells in the deep layers of cortex relative to GABA staining. Double-labeling with plasma and antibodies toward the calcium-binding proteins calbindin, parvalbumin and calretinin revealed that plasma-labeled cells commonly expressed calbindin. The autoantibody-positive cells expressed parvalbumin less commonly and calretinin only rarely. The distribution of GABAergic interneurons that colocalize one of these calcium-binding proteins is variable from one cortical area to the next, and certain interneuron types can colocalize more than one of the proteins [44-47]. Defelipe [48] provided a detailed description of the correspondence of specific interneuron types and immunoreactivity for a calcium-binding protein in the neocortex. In general, parvalbumin is associated with chandelier and large basket cells; calretinin is associated with bipolar, double-bouquet and Cajal-Retzius cells; and calbindin is associated with double-bouquet, neurogliaform, Martinotti and, in rare cases, Cajal-Retzius cells. This implies that the plasma autoantibodies affect neurons harboring calbindin more than those colocalizing the other calcium-binding proteins. However, it is not possible at this time to predict the specific functional significance of targeting these specific classes of interneurons. The question why some GABAergic interneurons are identified by the plasma autoantibodies and others is not very difficult to speculate on, given that the antigen is not known. However, recent neuronal transcriptome studies [49,50] have made it abundantly clear that tens to hundreds of proteins differentiate neuronal classes. Given the distinct morphological features of interneurons, it is highly likely that different classes of GABAergic neurons express different profiles of proteins and that one or more of these are being identified by the plasma autoantibodies.

Our finding that some children with autism produce autoantibodies directed against GABAergic neurons is all the more interesting, given the other indications that ASDs may be highly related to impaired GABAergic function. Previous studies have suggested that there is a depression of the GABAergic system, potentially due to altered GABA receptors and reduced GAD, in the etiology and maintenance of ASD [51-53]. It has also been proposed that suppression of GABAergic function in the brain of individuals with ASD may result in compensatory mechanisms and increased activation of additional GABAergic receptor subtypes [54]. The potential imbalance of excitatory and inhibitory neurotransmission in autism could influence cortical networks that control social behaviors and neuromodulatory systems [55]. Interestingly, a reduction in GABAergic activity is also capable of causing epilepsy, which has been reported in up to one-third of individuals with ASD [52,56-60]. While the participants in the present study were not assessed for epileptic seizure activity, this would be of great interest for future longitudinal studies.

In a study by Fatemi and colleagues [61], GAD, which converts glutamate to GABA and is a marker commonly used to identify GABAergic neurons, was found to be reduced by 48% to 61% in the parietal cortex and cerebellum in five individuals with autism relative to eight controls. More recent studies by Fatemi and colleagues [62] have found downregulated expression of various combinations of GABA_A _receptor subunits in the parietal cortex, cerebellum and superior frontal cortex in the brains of individuals with autism. These investigators also reported alterations of GABA_B _receptor subunit expression in the same areas of the brain [63]. Yip and colleagues [64,65] reported increased levels of GAD67 mRNA levels in cerebellar interneurons but decreased levels in Purkinje cells. Blatt *et al. *[66] examined the distribution and density of GABAergic, serotonergic, cholinergic and glutamatergic receptors in the hippocampus of individuals with ASD and typically developing controls. The only receptor system found to be significantly reduced in individuals with ASD was the GABAergic system [66]. Decreased GABA levels have also been reported in the platelets of children with ASD [67].

While it is intriguing that some children with autism demonstrate autoantibodies that are immunoreactive to central nervous system GABAergic neurons, it is not at all clear whether these antibodies play a pathophysiological role in the etiology of autism or are an epiphenomenon indicative of some other pathological process. In order for the systemic antibodies to interact with central nervous system neurons, they must be able to reach their antigenic targets. As antibodies do not readily cross the blood-brain barrier, a breach would have to occur to allow access. Cytokines in the peripheral circulation or cytokines produced by central nervous system cells are capable of altering the blood-brain barrier through endothelial cell activation [68]. Disturbances to the blood brain-barrier can be caused by a number of other factors, including extreme stress, subclinical infection and even nicotine or epinephrine exposure [69-71]. In studies by Diamond and colleagues [72,73] in which an animal model of systemic lupus erythematosus (SLE) was explored, antibodies directed against the *N*-methyl-D-aspartate receptor were able to enter the brain following exposure to either lipopolysaccharide or norepinephrine. These antibodies not only produced neuropathological changes in the hippocampus or amygdala, respectively, but also led to impaired memory function or increased anxiety.

If we presume that the anti-GABAergic neuron antibodies produced by children with autism can gain entry to the brain, there are several possible mechanisms through which they may influence cell function. First, autoantibodies may act as an agonist or a receptor ligand, causing excessive receptor stimulation. This phenomenon has been demonstrated in approximately 27% of patients with epilepsy and 30% of individuals with SLE, as well as in some patients with encephalitis, in whom various autoantibodies to the glutamate receptor have been described [74]. Further, both human and animal studies have demonstrated that the described glutamate receptor autoantibodies are pathologic, binding to neurons with a unique ability to activate their glutamate receptor antigen and leading to neuronal death, either through excitotoxicity or by complement fixation independent of receptor activation [74-76]. While a decreased number of neurons has been reported in the amygdala [77] and in the fusiform gyrus [78] of postmortem cases of autism, it is not yet clear whether there is a preferential loss of any particular category of neurons, such as GABAergic interneurons.

The second possibility for autoantibody-induced pathology is through antibody-mediated inhibition of a critical receptor, ligand or pathway. This would be similar to the pathologic mechanism through which the autoantibodies to thyroglobulin, thyroid peroxidase and/or the thyroid-stimulating hormone receptor function in Hashimoto's thyroiditis [79]. Whether through excitation or inhibition, autoantibody-mediated changes are capable of resulting in a number of permanent changes, including inhibition-excitation imbalance, altered neural circuitry and changes in receptor numbers and distribution. Finally, it is possible that the autoantibody directly induces cell and tissue destruction through complement activation such as that seen in patients with SLE [80]. Each of the above-discussed mechanisms, either individually or in concert, is a possibility with respect to the autoantibodies described herein. However, additional studies, including an animal model, are needed to determine the pathologic relationship between these autoantibodies and ASD.

## Conclusions

It is of great interest that autoantibodies from a subset of children with ASD are directed against GABAergic neurons with identical patterns of staining distributed throughout the central nervous system. This study raises several issues, including the identity of the target antigen that these antibodies recognize and the reason for the selectivity of this response. Knowledge about what differentiates immunopositive from immunonegative GABAergic neurons may provide further clues regarding the characteristics of the autoantigen and proteomic studies currently underway to determine the identity of the target protein. One additional point to address is whether the presence of these GABAergic neuron-specific autoantibodies is pathologically significant or merely an epiphenomenon. Ultimately, the potential mechanisms and/or exposures that lead to the generation of these autoantibodies, the timing at which they occur, and their pathologic significance require further consideration.

## Competing interests

The authors declare that they have no competing interests.

## Authors' contributions

JV, along with DGA, conceived of the study, participated in its design and coordination, and helped to draft the manuscript. SW carried out the investigation of plasma reactivity in the nonhuman primate tissue and helped to draft the manuscript. JB prepared tissues and helped with analysis of staining. CCR carried out the investigation of plasma reactivity in mouse tissue, processed and analyzed tissue double-labeled with plasma and anti-GABA, and participated in double-labeling with antibodies directed against calcium-binding proteins. VMC participated in colabeling experiments and manuscript preparation. PA helped with study design and preparation of the manuscript. All authors read and approved the final manuscript.
